# Partitioning of Proteins and Anti-Nutrients in Cassava (*Manihot esculenta* Crantz) Leaf Processing Fractions after Mechanical Extraction and Ultrafiltration

**DOI:** 10.3390/foods10081714

**Published:** 2021-07-23

**Authors:** Haimanot Hailegiorigs Ayele, Sajid Latif, Marieke E. Bruins, Joachim Müller

**Affiliations:** 1 Tropics and Subtropics Group, Institute of Agricultural Engineering, University of Hohenheim, 70599 Stuttgart, Germany; s.latif@uni-hohenheim.de (S.L.); joachim.mueller@uni-hohenheim.de (J.M.); 2 Wageningen Food & Biobased Research, Wageningen University & Research, 6708 WG Wageningen, The Netherlands; marieke.bruins@wur.nl

**Keywords:** cassava leaves, juice, press cake, mechanical pressing, ultrafiltration, mild processing, fractionation

## Abstract

Cassava plays a major role in improving food security and reducing malnutrition. The purpose of this study was to evaluate the influence of mechanical pressing coupled with ultrafiltration (UF) on the quality of different fractions of cassava leaves. Cassava leaves harvested from the greenhouse at the University of Hohenheim were passed through a mechanical screw press to extract the juice and separate the press cake. The juice was centrifuged and filtered to separate the sediment and clear supernatant. The clear supernatant was filtered using a 10 kDa UF system. The nutritional contents of the different fractions were analyzed at each processing step. The total phenolic content was significantly lower in the press cake that had a higher fiber and ash content. The juice and sediment fractions had higher crude protein and total phenolic content. Processing did not negatively affect the concentrations of essential amino acids except for tryptophan in the juice fraction. Non-protein nitrogen was mainly present in the UF permeate, illustrating the potential of UF for upgrading soluble protein fractions. The results indicated that the different fractions during processing could be a possible source of protein for food, feed (juice, sediment, and retentate), or fiber (press cake) for ruminant feed.

## 1. Introduction

Most of the global agricultural food production focuses on meeting energy requirements but the main cause of malnutrition is a shortage of proteins and vitamins in the diet [1]. The use of alternative nutritional plant sources like cassava increases access to a balanced diet by providing good amounts of protein and other essential nutrients without expanding production areas [2]. Cassava (*Manihot esculenta* Crantz), mainly cultivated for its root, is the world’s seventh most important crop in terms of production and it is the main food crop in many countries within the tropics [3]. Cassava plays a major role in improving food security and reducing malnutrition with its tolerance to extreme conditions, efficient energy production, and year-round availability [4,5,6]. The Cassava root is rich in carbohydrates but low in protein and vitamins [7]. Conversely, the leaf, a by-product of the cassava root harvest, is a good source of crude protein (CP) (11.8–38.1 g 100 g^−1^_DM_) and minerals [5,7,8,9,10] in addition to vitamins A, B1, B2, and C. Cassava leaves have the same yield as the roots in terms of fresh material [11,12]. Several researchers have confirmed the nutritional potential of cassava leaves as a complementary supplement to starchy diets if properly detoxified for both human consumption and animal feed [7,9,13,14]. Its nutritional content varies depending on the type of variety, cultivar, age of the plant, harvesting frequency, and processing methods [8,15].

Despite the huge potential of cassava leaves, they have not been widely incorporated into the food system as an alternative protein source [9]. The main reason for this is their high fiber content and the presence of anti-nutritional compounds including polyphenols, phytates, nitrates, saponins, oxalates, and cyanogenic compounds in the leaves [8,16,17,18]. To reduce the anti-nutritive properties and increase the nutritional value of the leaves, various processing techniques have been developed [19,20,21,22]. The most widely practiced processing techniques to use the leaves as food at the household level involves crushing, boiling, washing, and cooking [13]. However, the application of heat during leaf processing results in large losses of protein, thiamin, riboflavin, nicotinic acid, and vitamin C [21,22,23]. There is also a huge loss of amino acids, especially methionine and cysteine, during cassava leaf processing [23]. Protein extraction methods for cassava leaves with simultaneous detoxification and removal of anti-nutrients could be a good option for the efficient utilization of protein [7].

Several attempts have been made previously to extract protein from cassava leaves but the results indicated limitations in essential amino acids and protein recovery [7,15,16,20]. Generally, protein extraction from leaves involves four major steps: tissue disruption by mechanical treatment and separation of fiber; protein precipitation; protein concentration; and protein purification [15,17,24]. Screw pressing at both the lab and pilot scale is an efficient way to separate the fiber and extract the green juice from the leaves [25,26]. The green juice contains soluble proteins but also chlorophyll, chlorophyll-related proteins, membrane fragments, and other unwanted compounds [25]. The proteins in this fraction may be used for food or feed. The remaining cassava press cake produced during pressing can also be a good source of animal feed but for different applications as a more fiber rich feed with different types of protein [7]. From the green protein fraction, particulates and chlorophyll still need to be removed. This broadens the acceptance of the soluble protein from the plant juice [27]. The white protein, mainly RuBisCO (ribulose-1,5-bisphosphate-carboxylase/oxygenase), usually amounts to about 50% of the soluble proteins in the leaf. This value can vary between 40–60% depending on the extraction process and plant source type [17,25].

White protein can be further purified from the leaf concentrate by combining different methods such as pH or heat precipitation, organic solvent precipitation, and flocculation [28]. The combination of these steps allows for the removal of green fractions, grassy smells, green color, and bad tastes from the juice. This improves the functional properties of the white fraction [25]. The process to obtain a functional white fraction increases the possible use of existing bio-resources through milder extraction methods that preserves protein functionality and achieves food-grade products [24]. The white fraction from the extraction can be further filtered through membranes to obtain a protein product with high solubility and more concentrated protein isolates [25,29]. Separation through a membrane is very efficient in improving the sensory and nutritional properties while obtaining natural fresh-tasting, additive-free, and high-quality products. The separation process does not require heat or the use of chemical agents [30]. Ultrafiltration (UF) is a pressure-driven membrane process that can be used to fractionate mixtures based on different molecular weights [31].

Determination of the proximate composition and protein profiling during the processing of cassava leaves will provide insight into its further use as an alternative food crop [9]. Different methods can be applied to characterize proteins including the determination of amino acid composition and molecular weight. The commonly engaged technique to estimate the molecular weight is sodium dodecyl sulfate-polyacrylamide gel electrophoresis (SDS-PAGE) [9]. Leaf protein from many plant sources generally possesses a good amino acid composition but can lack methionine. Methionine and lysine in plant leaves are also easily destroyed during extraction, drying, and storage, resulting in the inferior nutritional value of the leaf protein [32]. One previous attempt was made by Castellanos et al. [33] to concentrate cassava leaf protein by employing UF but the nutritional value of the fractions during processing was not addressed. Therefore, the potential nutritional value indicated by the amino acid composition of different fractions of cassava leaves, which were gently extracted by mechanical pressing and UF without applying heat, was evaluated in this study.

## 2. Materials and Methods

### 2.1. Leaf Processing

**Figure 1:**
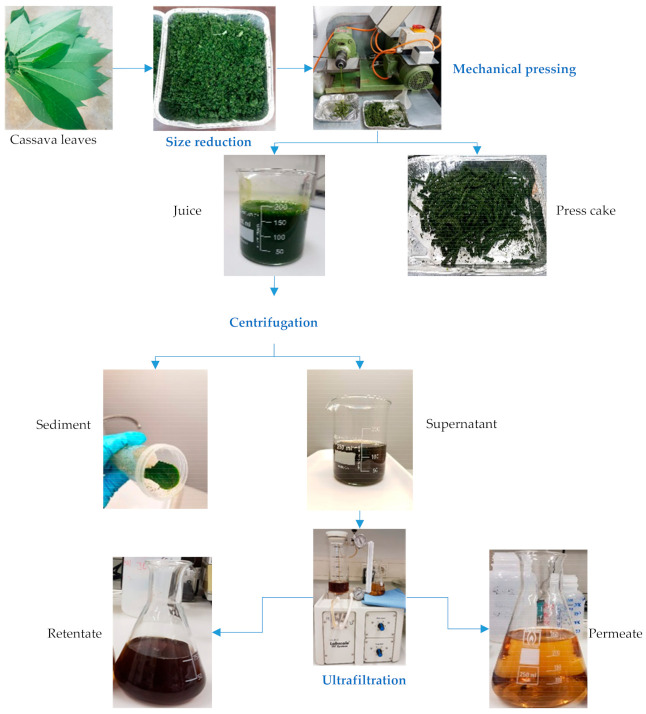
Cassava leaf processing via mechanical pressing and ultrafiltration.

Cassava leaves from six month-old plants grown in a greenhouse at the University of Hohenheim were harvested and taken to the laboratory. The leaves were ground in a kitchen chopper for 10 s at a speed of 10,200 rpm to reduce their size (Thermomix TM31, Vorwerk, Cloyes, France). The juice was extracted through a mechanical screw press with a 4 mm nozzle diameter and a screw speed of 18 rpm (CA59G, IBG Monforts Oekotec, Mönchengladbach, Germany) [26]. The leaf press cake was produced as a co-product. Separation of the supernatant and sediment from the juice fraction was achieved by centrifuging the juice at 6 °C and 13,500 rpm for 30 min (Z 326 K, Hermle Labortechnik GmbH, Wehingen, Germany). To avoid fouling of the membrane, the supernatant was filtered twice using 12 μm paper and then vacuum filtered through 1 μm filter paper (Figure 1).

### 2.2. Ultrafiltration Unit and Procedures

The molecular weight cut-off for the UF system was selected based on the SDS-PAGE result of the supernatant and previous reports of the cassava protein molecular weight of different varieties [9]. UF was run in a lab-scale tangential flow filter system (TFF 29751, Merck, Bangalore, India) equipped with a 10 kDa pellicone cassette composite regenerated cellulose membrane with 0.005 m^2^ filtration surface. To separate the retentate and permeate fractions, the processed clear cassava supernatant was passed through a membrane with 35 PSI transmembrane pressure (*TMP*), calculated as

(1) $$TMP=\frac{P_{f}+P_{r}}{2}-P_{p}$$

where *P_f_*, *P_r_*, and *P_p_* are the feed pressure, retentate pressure, and permeate pressure, respectively, given in PSI.

For UF, the volume concentration ratio (*VCR*) that relates to the concentration degree was calculated as follows [34]:

(2) $$VCR=\frac{V_{f}}{V_{r}}$$

where *V_f_* is the initial feed volume and *V_r_* is the final retentate volume, both in mm.

### 2.3. Mass Fractions and Dry Matter

The mass fraction *MF_n_* of the *n* = 6 fractions during processing was calculated according to Equation (3):

(3) $$MF_{n}=\frac{m_{n}}{m_{ini}}\cdot 100$$

where *m_n_* is the mass of fraction *n* and *m_ini_* is the initial mass of the sample.

The mass loss of processing methods was calculated by the difference from the initial weight. MF during processing was reported on a fresh matter (FM) basis.

Additionally, the samples were dried in an oven at 105 °C for 12 h [35] to calculate the dry matter (DM) content.

### 2.4. Crude Protein (CP)

The CP_n_ content of the six processing fractions was measured by the Kjeldahl method using the Kjeldahl analysis system (Vapodest 500, C. Gerhardt GmbH & Co. KG, Königswinter, Germany) according to the manufacturer’s guidelines. The CP_n_ content was calculated with a conversion factor of 6.25. The results are expressed in g per 100 g of DM (g 100 g^−1^_DM_).

### 2.5. Total Phenolic Content

**Figure A1:**
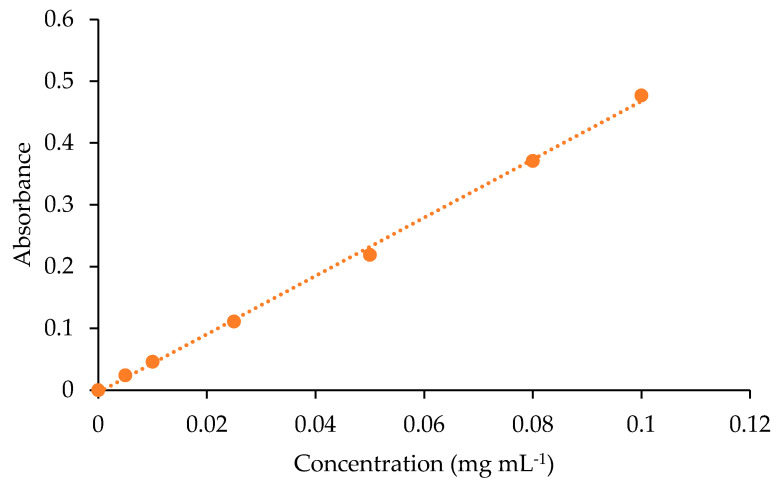
Calibration curve for TPC (R^2^ = 0.9985) based on the gallic acid equivalent, showing absorbance (ABS) as a function of the GAE equivalent (ABS = 4.715 GAE–0.004).

The total phenolic content (TPC) was determined by the Folin-Ciocalteu reagent method [36]. A sample of 1 g mL^−1^ was diluted in 3 mL 80% methanol, then mixed, and placed in a 60 °C water bath for 20 min. It was then centrifuged at 13,500 rpm for 10 min (Z 326 K, Hermle Labortechnik GmbH, Wehingen, Germany). The supernatant was transferred to a 10 mL volumetric flask where the residue was mixed again with 3 mL of 80% methanol and centrifuged. The supernatant was combined with the previous volume and was adjusted to 10 mL with 80% methanol. The extracted solution was kept at 4 °C until the analysis. The sample and standard were incubated for 2 h at room temperature in the dark using 80% methanol as a blank. The absorbance of the standards and the samples was measured using a UV-spectrophotometer (DR6000, Hach Lange, Düsseldorf, Germany) at 725 nm. The standard calibration curve was prepared with a gallic acid stock solution at 0.005 to 0.1 mg mL^−1^ (Figure A1). The TPC content was expressed as the gallic acid equivalent (GAE) (mg GAE g^−1^_DM_ for solid fractions; mg GAE mL^−1^ for liquid fractions).

### 2.6. Amino Acid Profile

The amino acid profile was determined by an amino acid analyzer system (Biochrom 30, Cambridge, England) according to the methods described in the European Commission regulation, number 152/2009 [37].

### 2.7. SDS-PAGE Analysis

Proteins from the clarified supernatant, retentate, and permeate were investigated through sodium dodecyl sulfate polyacrylamide gel electrophoresis (SDS-PAGE) [38]. A supernatant of 5 μL in addition to 1, 5, and 10 μL of retentate and permeate were TCA-precipitated, boiled in an SDS-loading buffer for 5 min, and loaded on a 12% acrylamide SDS-gel. A protein standard (NEB Biolabs, England) was also incorporated into the gel to determine the molecular weight of the bands. After the gel was run at 100 V for 60 min, proteins were fixed and stained with Coomassie Brilliant Blue in isopropanol–acetic acid. The gel was photographed after destaining.

### 2.8. Neutral Detergent Fiber, Acid Detergent Fiber, Acid Detergent Lignin, and Ash Content

The neutral detergent fiber (NDF), acid detergent fiber (ADF), and acid detergent lignin (ADL) were measured before and after pressing by AOAC [35] using the official method 973.18 (FibreBag Analysis System FBS6, Gerhardt GmbH & Co. KG, Königswinter, Germany). The ash content of the different fractions of the samples was determined by using a muffle furnace as described in AOAC [35] using the official method 923.03. The results are expressed on a DM basis (g 100 g^−1^_DM_).

### 2.9. Statistical Analysis

One-way analysis of variance (ANOVA) was conducted for the data obtained from three independent replicates, except for the amino acid profile (two replicates), using the SAS statistical software package (version 9.2, SAS Institute Inc., Cary, NC, USA). The statistically significant difference of the means was defined as *p* ≤ 0.05 using Tukey’s honest significant difference test.

## 3. Results

### 3.1. Mass Balance

The fresh cassava leaves, press cake, juice, sediment, retentate, and permeate had an average DM content of 22.6, 11.0, 57.4, 32.7, 7.0, and 5.0%, respectively.

**Figure 2:**
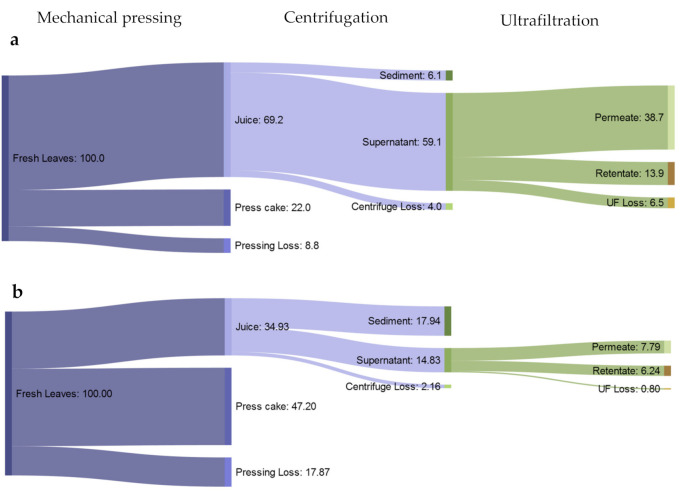
(**a**) Mass balance and (**b**) protein yield (%) of cassava leaf processing fractions after mechanical pressing, centrifugation, and ultrafiltration (UF) on a fresh matter basis.

After mechanical pressing, the press cake represented 22.0% of the initial mass on average and the juice 69.2%. This result was in line with previous results that also obtained 70% green juice from alfalfa leaves [39]. The average weight loss from the process of pressing and clarifying of the juice via centrifugation was 8.8% and 4.0%, respectively. By centrifugation and filtration, 6.1% sediment and 59.1% clear supernatant were obtained from cassava leaves (Figure 2a). After UF, a VCR of 4.1 was achieved with an average of 13.9% retentate and 38.7% permeate. The holdup volume (remaining liquid in the system’s piping and filter modules) will vary depending on the design of the piping, optimized total membrane area, and the design of the processing plant [40]. The average holdup volume from the current UF was 11.7% but this could be lowered by reducing the membrane area in the cassettes and using smaller UF systems with shorter piping designs [41]. The crude protein yield from pressing resulted in a higher amount of protein in the press cake (47.2%) in comparison with the juice (34.93%). After centrifugation and UF, the higher crude protein yield was found in the sediment (17.94%) and permeate (7.79%), respectively (Figure 2b).

### 3.2. Crude Protein

**Figure 3:**
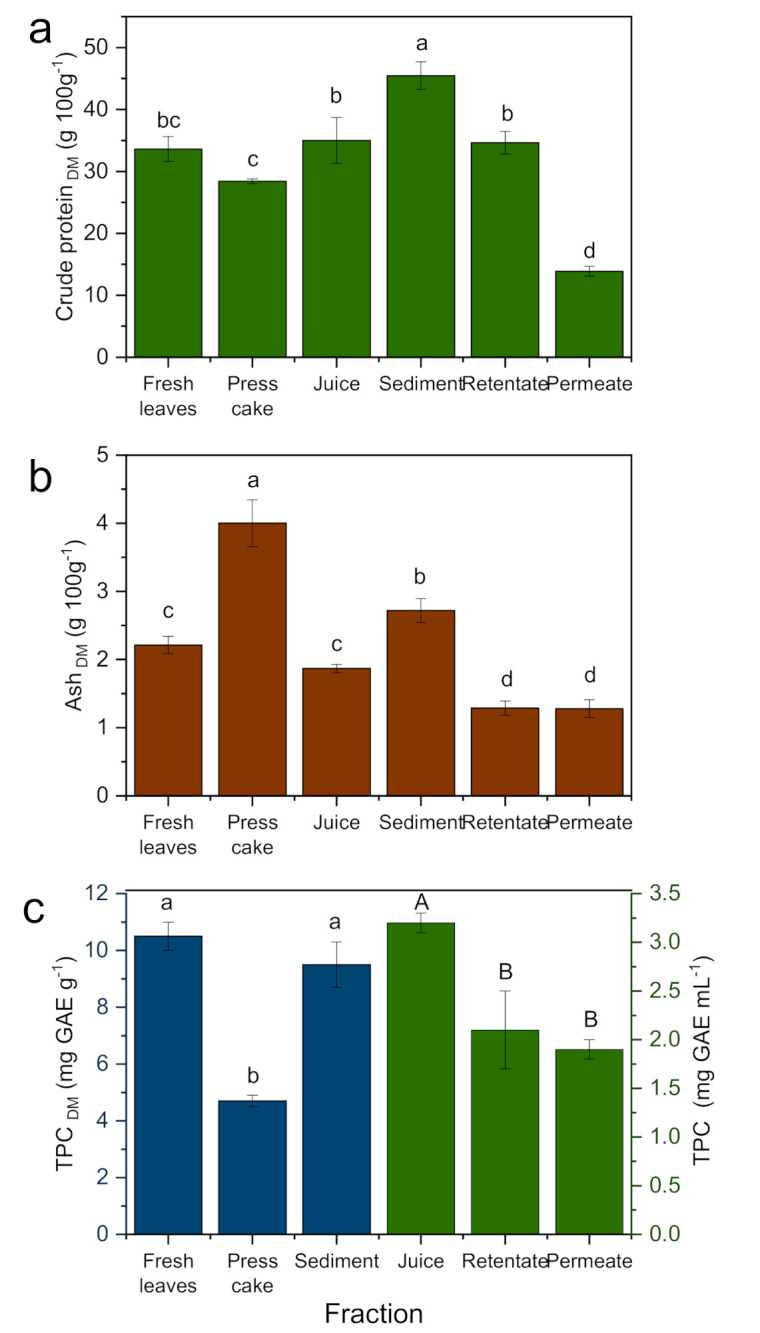
Nutritional content of cassava leaf processing fractions. (**a**) Crude protein CP_n_, (**b**) ash, and (**c**) total phenolic content (TPC). Means marked by different letters are statistically different (*n* = 3; Tukey test, *p* ≤ 0.05).

The CP content was significantly (*p* < 0.05) higher in the sediment (45.5 g 100 g^−1^_DM_) and lower in the permeate (13.9 g 100 g^−1^_DM_) fraction (Figure 3a). The CP content of the fresh leaves (33.6 g 100 g^−1^_DM_) was high due to the young age of the plants used in the experiment [10]. After mechanical pressing, the juice (35.0 g 100 g^−1^_DM_) had a significantly higher CP content than the press cake (28.4 g 100 g^−1^_DM_), which is similar to alfalfa pressing with a twin-screw extruder [39]. The high CP content in the sediment is in line with previous findings for sugar beet [24] and alfalfa leaves [39]. After centrifugation, some of the protein was still linked to the cell walls and/or was insoluble, and resulted in the higher CP content of the sediment fraction [39]. Teo et al. [42] stated that the protein content of cassava leaf protein concentrate might vary from 40 g to 70 g 100 g^−1^_DM_ based on the extraction method. Research conducted by Urribarrí et al. [17] on a cassava leaf protein extraction by enzymatic hydrolysis (cellulase and xylanase) demonstrated a higher CP content (36.4 g 100 g^−1^_DM_) from the fresh leaves (18.6 g 100 g^−1^_DM_). Another study by Castellanos et al. [33] also reported that the content of the cassava leaf protein after thermocoagulation (42.9 g 100 g^−1^_DM_) and UF using 40 kDa membrane (43.9 g 100 g^−1^_DM_) was high compared to the fresh leaves (22.0 g 100 g^−1^_DM_). In the current study, a CP content of 34.7 g 100 g^−1^_DM_ was concentrated in the retentate fraction. The different compositions of the individual fractions compared to other studies could be attributed to the different processing methods such as membrane cut-off or the variety and age of the plant [24,30]. UF was efficient in concentrating the crude protein that was indicated by the extremely low content in the permeate fraction. This is similar to reports for tea leaves with 20 kDa [43].

### 3.3. Ash

The percentage of ash, an indication of the total mineral content of the fractions, varied from 1.3 g 100 g^−1^_DM_ for the permeate to 4.0 g 100 g^−1^_DM_ for the press cake fraction during processing. This result is lower than that which was reported by Oresegun et al. [44] for different cassava varieties (2.7–5.6 g 100 g^−1^_DM_). The young age of the plant used in this experiment played a role in the low ash content (2.2 g 100 g^−1^_DM_) of the fresh leaves [10]. In general, cassava leaves have a lower ash content compared to other plant leaves used for protein extraction such as sugar beet (20 g 100 g^−1^_DM_) [24], *Solanum africana* (19.4 g 100 g^−1^_DM_), *Amaranthus hybridus* (22.3 g 100 g^−1^_DM_), *Telfaria occidentalis* (11.4 g 100 g^−1^_DM_), and *Vernonia amygdalina* (9.5 g 100 g^−1^_DM_) [45]. During mechanical pressing and centrifugation, the ash content in the press cake and sediment was significantly higher, while the reverse was true for the juice, permeate, and retentate fractions (Figure 3b). Other experiments suggested that the ash content of cassava leaves did not change after pounding, grinding, and cooking for 30–60 min [46], whereas the juice extraction of sugar beets with a screw press resulted in a higher ash content in the juice (31.0 g 100 g^−1^_DM_) than in the sediment (16.8 g 100 g^−1^_DM_) [24]. The low ash content in the permeate and retentate is an indication of the low mineral content in these fractions. This is in line with reports for the UF of *Atriplex lampa* leaves (10 kDa membrane) that resulted in a reduction of the ash content from 40% to 23% [31]. The current results differed from research conducted by Castellanos et al. [33] that stated that the ash content of cassava leaves was higher (6.0 g 100 g^−1^_DM_) after UF (40 kDa membrane) than in the fresh leaves (5.7 g 100 g^−1^_DM_). This deviation might be due to the difference in plant age, variety, and/or processing methods.

### 3.4. Total Phenolic Content

TPC, known for its ability to bind proteins and essential minerals, was reduced by 55.7% in the press cake fraction (Figure 3c). Research by Nur et al. [47] indicated that the TPC in cassava leaves could range from 9.1 to 11.5 mg GAE g^−1^ which is similar to the current finding for fresh leaves. After pressing and centrifuging, most of the TPC moved to the juice and sediment fractions (Figure 3c), indicating that TPC is soluble. This could furthermore be related to the positive correlation of TPC and protein content due to possible covalent bonds between them [48]. The molecular weight of tannins, a constituent of most of the TPC in cassava leaves, was greater than 5 kDa [49] and this led to a higher TPC content in the retentate fraction after UF. This was also true for the UF of *Castanea sativa* leaves using a 5 kDa membrane, resulting in a higher amount of TPC in the retentate fraction [50]. Other processing methods such as boiling cassava leaves can reduce TPC in the leaves by as much as 32% [51].

### 3.5. Amino Acid Profile

The amino acid profile showed equal or higher amounts in the solid fractions (press cake and sediment), while a minimal loss was observed in the liquid fractions (juice and retentate) (Table 1). The methionine and tryptophan content in the retentate were lower by 42% and 65%, respectively, whereas the lysine content was higher by 21% compared with the fresh leaves. The amino acid profile of the current retentate fraction was higher than that which was reported by Castellanos et al. [33] using a 40 kDa membrane. The reasons for this include differences in variety, age of the plant, and membrane size. Eggum [52] reported that only 60% of the methionine was biologically available in boiled cassava leaves. Other authors have also stated that cooking cassava leaves even for a short time could result in a reduction of essential amino acids [7,21,23]. Both processing methods resulted in a good amino acid profile except for the permeate [53]. The low total amino acid content (36 g 100 g^−1^ protein on DM) in the permeate fraction (Table 1) is an indicator that not all nitrogen in the fraction is from the protein. Apart from the protein or amino acids, the nitrogen might derive from nitrogenous compounds such as amines, nucleic acids, ammonia, urea, nitrites, nitrates, phospholipids, or nitrogenous glycosides [54]. This indicates the effectiveness of the ultrafiltration system in concentrating the essential amino acids in the retentate fraction and other nitrogenous compounds in the permeate. The total amino acid profile of cassava leaves, press cake, juice, sediment, and retentate indicated that the CP is almost equal to the true protein. The amount of tryptophan, threonine, valine, methionine, isoleucine, leucine, histidine, lysine, cysteine, and aromatic amino acids (Phenylalanine + tyrosine) in all the fractions except permeate were similar or higher than what was recommended by FAO/WHO/UNU [53]. The available literature also suggests that apart from lower methionine and lysine contents, the essential amino acid profile of cassava leaves is similar to those of fish, milk, soybeans, eggs, and cheese [9].

### 3.6. SDS-PAGE Analysis

**Figure 4:**
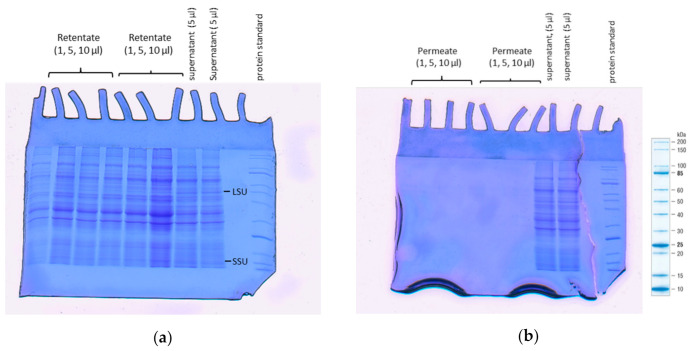
Protein electrophoretic patterns of the supernatant in comparison with (**a**) the retentate and (**b**) the permeate fraction of cassava leaves. RuBisCo large (LSU) and small (SSU) subunits.

A large variety of bands ranging from 10–200 kDa that show large (53 kDa) and small (12 kDa) RuBisCo subunits were generated in the retentate and supernatant fractions (Figure 4a). The similar patterns indicate that the cassava leaf protein profile of the retentate was not changed after UF. The molecular weight range on the SDS-PAGE of the current result was higher than that which was described by Popoola [9] (14–100 kDa). This variation could be caused by the different protein extraction processes and the variety used in the experiment [9]. No visible band was seen on the permeate fraction (Figure 4b). This illustrates that after UF, the protein fraction was concentrated in the retentate.

### 3.7. Neutral Detergent Fiber (NDF), Acid Detergent Fiber (ADF), and Acid Detergent Lignin (ADL)

Cassava leaves have been used as a feed supplement in combination with other feed materials due to its low fiber content [55,56]. NDF (cellulose, hemicellulose, and lignin), ADF (lignin and cellulose), and ADL (lignin) are the different fiber fractions that define the quality of the feed product [57]. About 65–75% of the variable costs in animal production are directly linked to feeding costs [58]. After mechanical pressing, all three attributes were significantly (*p <* 0.05) higher in the press cake while the reverse was true for the juice fraction (Table 2). The NDF and ADF reported for fresh leaves in the current experiment were fairly similar to the report by Ravindran and Ravindran [10]. Increasing the NDF and ADF proportion in the press cake will improve the feed quality of cassava leaves for ruminants [15] but it has a negative effect for humans and monogastric animals [59]. The low amount of fiber in the juice fraction establishes it a potential feed for monogastric animals and food due to the separation of the protein from the fiber [55].

## 4. Conclusions

Avoiding heat application when processing cassava leaves via mechanical pressing and ultrafiltration (UF) resulted in highly nutritious fractions. UF was effective in concentrating cassava leaf protein in the retentate fraction without changing the protein profile of the leaf, although the total phenolic content (TPC) was high. The results indicated that cassava leaves, sediment, and the juice fraction could be potentially used as a source of protein with the limitations of a higher TPC. The press cake co-fraction could be a good source for ruminant feeds with a good fiber and a low phenolic content. The protein of the different leaf fractions established them as a possible source for complementing other conventional foods and feed. The amino acid profile of the different fractions apart from the permeate suggested the potential benefits of cassava leaves. These findings support the use of cassava leaves as a food security crop with balanced nutrients. Further studies should be conducted to address the cyanogenic potential and other anti-nutritional contents of the fractions. In addition, the optimization of the UF process, considering the transmembrane pressure, pH, volume concentration ratio, and temperature, will play an important role in the quality of the protein concentrate obtained from cassava leaves via pressing and UF.

## Figures and Tables

**Table 1:** Amino acid profile of leaf fractions after mechanical pressing and ultrafiltration (UF).

Amino Acid	Fractions						Recommended Amount *
	Fresh Leaves	Press Cake	Juice	Sediment	Retentate	Permeate	
Tryptophan	2.0 ± 0.1 ^a^	2.2 ± 0.1 ^a^	0.5 ± 0.0 ^bc^	2.5 ± 0.3 ^a^	0.7 ± 0.1 ^b^	0.0 ^c^	0.6
Threonine	4.5 ± 0.2 ^a^	4.7 ± 0.0 ^a^	4.8 ± 0.3 ^a^	4.8 ± 0.3 ^a^	5.2 ± 0.6 ^a^	1.4 ± 0.0 ^b^	2.3
Valine	5.7 ± 0.3 ^a^	6.1 ± 0.3 ^a^	5.8 ± 0.2 ^a^	6.3 ± 0.0 ^a^	6.5 ± 0.0 ^a^	2.1 ± 0.2 ^b^	3.9
Methionine	1.9 ± 0.1 ^ab^	1.9 ± 0.1 ^ab^	1.4 ± 0.0 ^bc^	2.1 ± 0.0 ^a^	1.1 ± 0.3 ^c^	0.0 ^d^	1.6
Isoleucine	4.7 ± 0.2 ^a^	5.2 ± 0.3 ^a^	4.6 ± 0.2 ^a^	5.3 ± 0.0 ^a^	5.0 ± 0.6 ^a^	1.4 ± 0.0 ^b^	3.0
Leucine	8.9 ± 0.2 ^ab^	9.9 ± 0.3 ^ab^	8.9 ± 0.2 ^ab^	10.3 ± 0.2 ^a^	8.3 ± 1.1 ^b^	1.4 ± 0.0 ^c^	5.9
Phenylalanine	5.8 ± 0.4 ^bc^	6.7 ± 0.1 ^ab^	5.8 ± 0.2 ^bc^	7.1 ± 0.1 ^a^	5.0 ± 0.6 ^c^	1.4 ± 0.0 ^d^	N/A
Histidine	2.2 ± 0.1 ^a^	2.2 ± 0.1 ^a^	2.1 ± 0.0 ^a^	2.2 ± 0.0 ^a^	1.8 ± 0.0 ^b^	0.0 ^c^	1.5
Lysine	6.3 ± 0.3 ^a^	6.4 ± 0.3 ^a^	6.5 ± 0.2 ^a^	6.4 ± 0.1 ^a^	7.9 ± 1.0 ^a^	2.8 ± 0.0 ^b^	4.5
Aspartic acid	10.1 ± 0.3 ^a^	9.7 ± 0.3 ^ab^	10.2 ± 0.3 ^a^	10.1 ± 0.5 ^a^	11.4 ± 0.4 ^a^	5.6 ± 2.0 ^b^	N/A
Glutamic acid	12.6 ± 0.3 ^a^	11.5 ± 0.4 ^a^	12.6 ± 0.3 ^a^	12.1 ± 0.3 ^a^	14.2 ± 1.6 ^a^	7.1 ± 2.0 ^b^	N/A
Alanine	6.6 ± 0.2 ^a^	6.9 ± 0.2 ^a^	7.0 ± 0.2 ^a^	7.1 ± 0.2 ^a^	7.4 ± 1.0 ^a^	3.5 ± 1.0 ^b^	N/A
Tyrosine	3.5 ± 0.3 ^a^	3.6 ± 0.1 ^a^	4.3 ± 0.0 ^a^	4.1 ± 0.1 ^a^	4.1 ± 0.6 ^a^	1.4 ± 0.0 ^b^	N/A
Serine	4.2 ± 0.0 ^a^	4.4 ± 0.1 ^a^	4.4 ± 0.2 ^a^	4.3 ± 0.3 ^a^	5.4 ± 0.6 ^a^	1.4 ± 0.0 ^b^	N/A
Glycine	5.8 ± 0.2 ^a^	6.2 ± 0.2 ^a^	6.1 ± 0.2 ^a^	6.5 ± 0.2 ^a^	5.9 ± 0.6 ^a^	2.1 ± 0.1 ^b^	N/A
Cysteine	0.8 ± 0.0 ^d^	0.8 ± 0.0 ^d^	1.2 ± 0.0 ^c^	0.7 ± 0.0 ^d^	1.8 ± 0.0 ^a^	1.4 ± 0.0 ^b^	0.6
Arginine	5.9 ± 0.2 ^ab^	5.7 ± 0.1 ^ab^	6.1 ± 0.2 ^ab^	6.5 ± 0.2 ^a^	5.0 ± 0.2 ^b^	1.4 ± 0.0 ^c^	N/A
Proline	5.1 ± 0.1 ^a^	5.7 ± 0.0 ^a^	5.0 ± 0.0 ^a^	5.5 ± 0.1 ^a^	5.0 ± 0.2 ^a^	1.4 ± 0.0 ^b^	N/A
AAA	9.2	10.3	10.1	11.2	9.2	2.8	3.8
Total amino acid	96.4	99.6	97.3	103.8	101.5	36.0	N/A

Values are expressed in g 100 g^−1^ protein of each fraction on a DM basis. Abbreviations: AAA = aromatic amino acids (phenylalanine + tyrosine) and N/A = data not available. * FAO/WHO/UNU indicate reference values [53]. Means in lines marked by different letters are statistically different (*n* = 2; Tukey test, *p* ≤ 0.05).

**Table 2:** Neutral detergent fiber (NDF), acid detergent fiber (ADF), and acid detergent lignin (ADL) of cassava leaf fractions during mechanical pressing.

Fractions	NDF (g 100 g^−1^_DM_)	ADF (g 100 g^−1^_DM_)	ADL (g 100 g^−1^_DM_)
Fresh leaves	19.5 ± 3.0 ^b^	13.8 ± 0.3 ^b^	1.4 ±0.1 ^b^
Press cake	28.0 ± 0.5 ^a^	25.3 ± 0.2 ^a^	4.0 ± 0.4 ^a^
Juice	9.3 ± 2.1 ^c^	1.0 ± 0.0 ^c^	0.0 ^c^

Means in columns marked by different letters are statistically different (*n* = 3; Tukey test, *p* ≤ 0.05).

## Data Availability

The data presented in this study are available on request from the corresponding author. The data are not publicly available due to privacy.
